# Life expectancy at birth in Duchenne muscular dystrophy: a systematic review and meta-analysis

**DOI:** 10.1007/s10654-020-00613-8

**Published:** 2020-02-27

**Authors:** Erik Landfeldt, Rachel Thompson, Thomas Sejersen, Hugh J. McMillan, Janbernd Kirschner, Hanns Lochmüller

**Affiliations:** 1 Department of Women’s and Children’s Health, Karolinska Institutet, Tomtebodavägen 18 A, 17177 Stockholm, Sweden; 2 Children’s Hospital of Eastern Ontario Research Institute, University of Ottawa, Ottawa, Canada; 3 Karolinska University Hospital, Astrid Lindgren Children’s Hospital, Stockholm, Sweden; 4 Department of Neuropediatrics and Muscle Disorders, Medical Centre, Faculty of Medicine, University of Freiburg, Freiburg, Germany; 5 Department of Neuropediatrics, Faculty of Medicine, University Hospital Bonn, Bonn, Germany

**Keywords:** Survival, Prognosis, Mortality, Mechanical ventilation

## Abstract

Several studies indicate that prognosis for survival in Duchenne muscular dystrophy (DMD) has improved in recent decades. However, published evidence is inconclusive and some estimates may be obsolete due to improvements in standards of care, in particular the routine use of mechanical ventilatory support in advanced stages of the disease. In this systematic review and meta-analysis (PROSPERO identifier: CRD42019121800), we searched MEDLINE (through PubMed), CINAHL, Embase, PsycINFO, and Web of Science for studies published from inception up until December 31, 2018, reporting results of life expectancy in DMD. We pooled median survival estimates from individual studies using the median of medians, and weighted median of medians, methods. Risk of bias was established with the Newcastle–Ottawa Scale. Results were stratified by ventilatory support and risk of bias. We identified 15 publications involving 2662 patients from 12 countries from all inhabited continents except Africa. Median life expectancy without ventilatory support ranged between 14.4 and 27.0 years (pooled median: 19.0 years, 95% CI 18.0–20.9; weighted pooled median: 19.4 years, 18.2–20.1). Median life expectancy with ventilatory support, introduced in most settings in the 1990s, ranged between 21.0 and 39.6 years (pooled median: 29.9 years, 26.5–30.8; weighted pooled median: 31.8 years, 29.3–36.2). Risk of bias had little impact on pooled results. In conclusion, median life expectancy at birth in DMD seems to have improved considerably during the last decades. With current standards of care, many patients with DMD can now expect to live into their fourth decade of life.

## Introduction

Duchenne muscular dystrophy (DMD) is an X-linked recessive and severely debilitating neuromuscular disease with an estimated incidence of about 1 in 3800–6300 live male births [1, 2]. DMD is characterized by progressive muscle degeneration caused by deficiency or complete absence of dystrophin protein, resulting in delayed motor milestones, loss of independent ambulation, and fatal cardiac and respiratory complications [3]. Per current clinical management guidelines [4], mechanical ventilatory support is usually introduced sometime during the second to third decade of the patient’s life, initially at night to treat sleep-related breathing disorders and hypoventilation. Yet, as the respiratory muscles continue to deteriorate, all patients eventually need assistance to breathe also during the daytime to survive [5]. Due to the morbidity and mortality caused by the disease, DMD has been shown to be associated with a substantial burden on affected patients [6], informal caregivers [7], and society [8].

Reports from several studies indicate that life expectancy at birth in DMD has improved in recent decades [9–13]. However, estimates vary markedly between samples, and, to date, no study has reviewed the body of literature on life expectancy in this indication. In addition, some published estimates may no longer be relevant due to improvement in standards of care, in particular the routine use of mechanical ventilatory support in advanced disease stages [14]. When healthcare practitioners communicate with families of newly diagnosed patients, there may thus exist some uncertainty regarding current prognosis for survival. To help bridge this evidence gap, the objective of our study was to conduct a systematic review and meta-analysis of life expectancy at birth in DMD.

## Methods

This systematic review and meta-analysis was registered at the International prospective register of systematic reviews (PROSPERO) (identifier: CRD42019121800), and conducted and reported in accordance with the Preferred Reporting Items for Systematic Reviews and Meta-Analyses (PRISMA) statement and the Meta-analysis Of Observational Studies in Epidemiology (MOOSE) checklist [15].

### Search strategy and selection criteria

On January 9, 2019, we searched MEDLINE (through PubMed), CINAHL, Embase, PsycINFO, and Web of Science for studies published from inception up until December 31, 2018, reporting results of life expectancy at birth in DMD without language restrictions. In PubMed, we used the search term ((“Muscular Dystrophy, Duchenne”[Mesh] OR “Duchenne muscular dystrophy”[TIAB]) AND (“Mortality”[Mesh] OR “Life Expectancy”[Mesh] OR “Survival”[Mesh] OR “Mortality”[TIAB] OR “Life Expectancy”[TIAB] OR “Survival”[TIAB])). In CINAHL, we used the search term ((TX Duchenne muscular dystrophy) AND ((TX mortality OR (TX life expectancy) OR (TX survival))). In Embase, we used the search term ((‘Duchenne muscular dystrophy’) AND (‘mortality’ OR ‘life expectancy’ OR ‘survival’)). In PsycINFO, we used the search term ((Duchenne muscular dystrophy AND (mortality OR life expectancy OR survival)).af). In Web of Science, we used the search term (TOPIC: (Duchenne muscular dystrophy) AND (TOPIC: (mortality) OR TOPIC: (life expectancy) OR TOPIC: (survival)). For studies including different indications, we required that results were reported separately for patients with DMD.

### Screening, data extraction, and synthesis

Two independent investigators (EL and RT) initially screened article titles and abstracts for eligibility, and subsequently reviewed full-text versions of selected records. The reasons for article exclusion were recorded and potential disagreements were specified to be resolved by the involvement of a third investigator (HL). Risk of bias was established with the Newcastle–Ottawa Scale [16]. To ascertain selection, we required patients to be diagnosed with DMD, that the diagnosis was confirmed via genetic analysis and/or muscle biopsy (or electromyography, muscle biopsy, and/or creatine kinase levels for cases discovered prior to 1988), that the sample was not restricted in terms of cause of death or underlying mutation (or other markers limiting representativeness), and adjustment for any concurrent illnesses associated with excess mortality at start of study (assessment of non-exposed was not applicable); to ascertain comparability, we required details of the use of ventilatory support in the sample population (assessment of adjustment for sex was not applicable); and to ascertain outcome, we required that all deaths were clinically confirmed, a minimal follow-up of 5 and 10 years for prospective studies of patients with and without ventilatory support, respectively, and that < 25% of the total sample were lost to follow-up during the study period.

For all articles included in the review, the following data were extracted: Author, year of publication, setting, design, sample, and median survival (i.e., the amount of time after which 50% of the patients have died) from birth. We did not extract estimates of mean age at death, as these measures fail to account for censoring. Data presented in graphs were extracted using a graph digitizing software (DigitizeIt).

Estimates of median survival from individual studies were pooled using the median of medians, and weighted median of medians (with weights proportional to the number of patients in the study and normalized to sum to 1), as proposed by McGrath et al. [17]. These methods have been shown to perform better than transformation-based approaches, where the sample mean and its sampling variance are estimated from median data [17]. We constructed approximate 95% confidence intervals (CIs) for the pooled median using the $\frac{1}{2} - { \hbox{min} }\left\{ {\frac{1}{2},\frac{{Z_{0.025} }}{2\surd k}} \right\}$ quantile of the observed study medians as the lower limit, and the $\frac{1}{2} + { \hbox{min} }\left\{ {\frac{1}{2},\frac{{Z_{0.025} }}{2\surd k}} \right\}$ quantile as the upper limit, where $k$ is the number of medians and $Z_{0.025}$ is the 0.975 quantile of the standard normal distribution. Approximate 95% CIs for weighted pooled medians were calculated in R using the wtd.quantile function in the Hmisc package as described by McGrath et al. [17].

We performed stratified analysis by ventilatory support (i.e., mechanical non-invasive ventilation or tracheotomy, as reported by the identified publications) and low versus high risk of bias. Temporary ventilatory support (e.g., mechanical ventilation via endotracheal intubation) for patients having received general anesthetic for a surgery or treatment of a reversible respiratory infection would not meet criteria for having received ventilatory support. Similarly, patients treated with non-invasive overnight bi-level positive airway pressure (BiPAP) or continuous positive airway pressure (CPAP) for the treatment of nocturnal hypoventilation and/or obstructive sleep apnea would also not be included in this group. Additionally, we also explored the impact on pooled estimates of excluding countries represented in multiple studies.

## Results

**Fig. 1:**
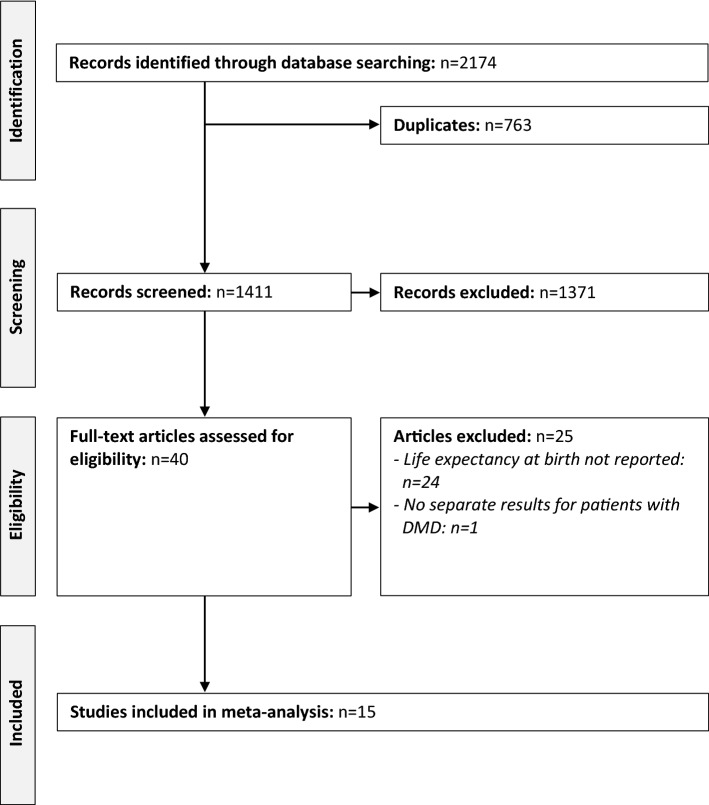
Study selection for meta-analysis. *Note*: *DMD* Duchenne muscular dystrophy

The systematic review resulted in the identification of 2174 publications (Fig. 1). Of these, 763 were duplicates, 1371 were excluded following title and abstract screening, and 40 were selected for full-text review. Finally, 14 articles [9–13, 18–26] and 1 editorial commentary (reporting previously unpublished data) [27] were considered for the meta-analysis. Summary data of the included publications are presented in Table 1. Identified studies encompassed 2662 patients with DMD from 12 countries from all inhabited continents except Africa. As some countries were represented by more than one study each, we cannot rule out that a proportion of patients might have been included more than once. As expected considering the outcome of interest, most articles (67%, 10 of 15) described results from retrospective chart reviews.

**Table 1:** Characteristics of included studies

References	Setting	Design	Sample	Life expectancy results	Risk of bias^a^
Bach et al. [24]	USA	Prospective observational study	101 patients with DMD with end-stage respiratory muscle failure receiving NIV treated at the University Hospital in Newark (New Jersey, USA) July 2010	Median survival (total sample; n = 101): 30.6 years	Selection: ◊◊◊◊ Comparability: ◊◊ Outcome: ◊◊◊
Eagle et al. [9]	UK	Retrospective chart review	165 patients with DMD treated at the Newcastle Muscle Centre (Newcastle upon Tyne, UK) who died between 1967 and 2002, and 18 living patients	Median survival (died in the 1960s without ventilatory support; n = 9): 14.4 years Median survival (died in the 1970s without ventilatory support; n = 49): 18.0 years Median survival (died in the 1980s without ventilatory support; n = 68): 18.7 years Median survival (died after 1990 without ventilatory support; n = 33): 19.1 years Median survival (died after 1990 with ventilatory support; n = 24): 26.2 years	Selection: ◊◊◊◊ Comparability: ◊◊ Outcome: ◊◊◊
Eagle et al. [20]	UK	Retrospective chart review	75 patients with DMD treated at the Newcastle Muscle Centre (Newcastle upon Tyne, UK) born between 1970 and 1990	Median survival (spinal surgery and ventilatory support; n = 27): 30.0 years Median survival (ventilatory support; n = 13): 22.2 years Median survival (no spinal surgery or ventilatory support; n = 35): 17.1 years	Selection: ◊◊◊◊ Comparability: ◊◊ Outcome: ◊◊◊
Gomez-Merino et al. [23]	USA	Retrospective chart review	57 patients with DMD with non-invasive IPPV, of which 14 subsequently underwent tracheotomy, treated at a university hospital neuromuscular disease clinic since 1983	Median survival (total sample; n = 57): 28.9 years	Selection: ◊◊◊◊ Comparability: ◊◊ Outcome: ◊◊◊
Gordon et al. [21]	Canada	Retrospective chart review	44 patients with DMD treated at the Pediatric Neurology Division at Dalhousie University in Halifax (Nova Scotia, Canada) born between 1963 and 2006 who had received at least 1 year of corticosteroid therapy. One patient was mechanically ventilated	Median survival (bisphosphonate treatment; n = 16): 27.0 years Median survival (no bisphosphonate treatment; n = 28): 21.0 years	Selection: ◊◊◊◊ Comparability: ◊◊ Outcome: ◊◊◊
Ishikawa et al. [10]	Japan	Retrospective chart review	187 patients with DMD treated at a medical institution in Yakumo (Hokkaido, Japan) between 1964 and 2010	Median survival (died between 1964 and 1984 without ventilatory support; n = 56): 18.1 years Median survival (died between 1984 and 1991 without ventilatory support; n = 11): 17.3 years Median survival (died between 1984 and 1991 with tracheotomy; n = 24): 29.7 years Median survival (died after 1991 without ventilatory support; n = 8): 21.9 years Median survival (died after 1991 with NIV; n = 88): 39.6 years	Selection: ◊◊◊◊ Comparability: ◊◊ Outcome: ◊◊◊
Kennedy et al. [22]	Australia	Retrospective chart review	38 patients with DMD treated at the Orthopaedic Clinic at the Women’s and Children’s Hospital or the Muscular Dystrophy Clinic at the Regency Park Centre for Young Disabled (Adelaide, South Australia) between 1960 and 1993	Median survival (spinal surgery; n = 17): 19.0 years. Median survival (no spinal surgery; n = 21): 19.0 years	Selection: ◊◊◊◊ Comparability: ◊◊ Outcome: ◊◊◊
Kieny et al. [11]	France	Retrospective chart review	119 adult patients with DMD treated at the AFM Yolaine de Kepper centre (Saint-Georges-Sur-Loire, France) between 1981 and 2011	Median survival (born before 1970 with/without ventilatory support; n = 43): 25.8 years Median survival (born after 1970 with/without ventilatory support; n = 76): 41.0 years Median survival (ventilatory support; n = 77): 36.2 years Median survival (no ventilatory support; no = 42): 22.2 years	Selection: ◊◊◊◊ Comparability: ◊◊ Outcome: ◊◊◊
Kohler et al. [26]	Switzerland	Prospective observational study	43 patients with DMD residing at the Mathilde-Escher-Heim center (Zurich, Switzerland) between 1999 and September 2006. 22 patients received long-term assisted mechanical ventilation for chronic respiratory failure	Median survival (total sample; n = 43): 35.0 years	Selection: ◊◊◊◊ Comparability: ◊◊ Outcome: ◊◊ (follow-up was too short)
Passamano et al. [12]	Italy	Retrospective chart review	516 patients with DMD treated at the Centre of Cardiomyology and Medical Genetics of the Second University of Naples (Naples, Italy) between 1961 and 2006	Median survival (born in the 1960s; n = NR): 18.0 years Median survival (born in the 1970s; n = NR): 22.1 years Median survival (born in the 1980s; n = NR): 28.0 years	Selection: ◊◊◊◊ Comparability: ◊ (ventilatory support details NR) Outcome: ◊◊◊
Rall et al. [19]	Germany	Retrospective chart review; cross-sectional observational study	67 patients with DMD born between 1970 and 1980 treated at the Department of Human Genetics, University of Würzburg (Würzburg, Germany)	Median survival (total sample; n = 67): 24.0 years. Median survival (no ventilatory support; n = 22): 19.0 years. Median survival (ventilatory support; n = 44): 27.0 years	Selection: ◊◊◊◊ Comparability: ◊◊ Outcome: ◊◊◊
San Martín Peñailillo et al. [18]	Chile	Retrospective observational study	462 patients with DMD treated at the Teletón Institute of Santiago (Santiago, Chile) between 1993 and 2013	Median survival (total sample; n = 462): 20.3 years. Median survival (low socio-economic status; n = 351): 19.0 years. Median survival (medium socio-economic status; n = 82): 23.3 years. Median survival (high socio-economic status; n = 15): 22.7 years	Selection: ◊◊◊ (uncertain diagnosis) Comparability: ◊ (ventilatory support details NR) Outcome: ◊◊◊
Toussaint et al. [25]	Belgium	Prospective observational study	42 patients with DMD with nasal IPPV receiving mouth IPPV since end-diurnal hypercapnia treated at the Neuromuscular Excellency Centre (Brussels, Belgium) between 1996 and 2005	Median survival (total sample; n = 42): 31.0 years	Selection: ◊◊◊◊ Comparability: ◊◊ Outcome: ◊◊◊
van den Bergen et al. [13]	The Netherlands	Cross-sectional observational study	293 patients with DMD born between 1961 and 1974 (pathway for identification NR), and 336 patients with DMD born between 1980 and 2006 registered in the Dutch Dystrophinopathy Database	Median survival (born 1961–1974; n = 293): 18.0 years Median survival (born 1980–2006; n = 336): 29.0 years	Selection: ◊◊◊◊ Comparability: ◊ (ventilatory support details NR) Outcome: ◊◊ (caregiver-reported)
Yasuma et al. [27]	Japan	Retrospective chart review	80 patients with DMD who died between 1980 and 1995, and 19 living patients with IPPV	Median survival (no ventilatory support; n = 65): 20.1 years. Median survival (NPV support; n = 7): 21.0 years. Median survival (IPPV support; n = 27): 30.4 years	Selection: ◊◊◊◊ Comparability: ◊◊ Outcome: ◊◊◊

^a^Assessed with the Newcastle–Ottawa Scale

Maximum score: ◊◊◊◊ for selection, ◊◊ for comparability, and ◊◊◊ for outcome

*NIV* non-invasive ventilation, *IPPV* intermittent positive-pressure ventilation. *NPV* negative pressure ventilation, *NR* Not reported, *DMD* Duchenne muscular dystrophy

**Fig. 2:**
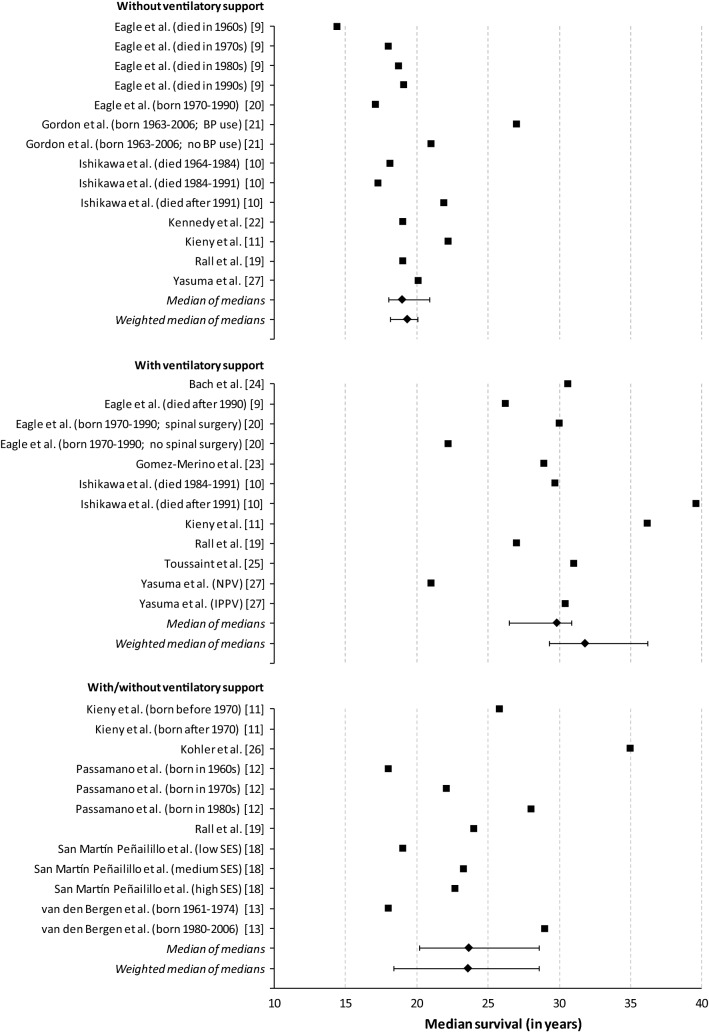
Meta-analysis of life expectancy at birth in DMD, *Note*: *BP* Bisphosphonate, *SES* socio-economic status, *DMD* Duchenne muscular dystrophy, *IPPV* intermittent positive-pressure ventilation, *NPV* Negative pressure ventilation. In the absence of data, the sample studied by Passamano et al. [12] was assumed to be uniformly distributed across listed strata when calculating pooled estimates

Across included publications, median life expectancy at birth in patients with DMD who did not receive ventilatory support ranged between 14.4 and 27.0 years (Fig. 2). The lowest estimate was derived from a sample of 9 UK patients who died in the 1960s [9], and the highest for 16 Canadian patients treated with bisphosphonates taken from a cohort born between 1963 and 2006 [21]. For this non-ventilated patient population, the pooled median life expectancy at birth was estimated at 19.0 years (95% CI 18.0–20.9) and weighted pooled median at 19.4 years (18.2–20.1).

Median life expectancy at birth in patients with DMD who received ventilatory support was notably higher, ranging between 21.0 and 39.6 years. The lowest estimate was based on data from a subgroup of 7 Japanese patients treated with negative pressure ventilation with cuirass respirator who died 1980–1995 [27], and the highest from 88 Japanese patients receiving non-invasive ventilation since 1991 treated at a medical institution between 1964 and 2010 [10]. The pooled median and weighted pooled median life expectancy at birth for these ventilated patients with DMD were estimated at 29.9 (95% CI 26.5–30.8) and 31.8 years (29.3–36.2), respectively.

Estimates derived from samples including both non-ventilated and ventilated patients with DMD ranged between 18.0 and 41.0 years. The lowest estimate was obtained from two sources: a sample of Italian patients (number of patients not reported) born in the 1960s [12], and 293 Dutch patients born 1961–1974 [13]. The highest estimate was based on a sample of 76 French patients born after 1970 [11]. For this mixed patient population, the pooled median was estimated at 23.7 years (95% CI 20.2–28.6) and weighted pooled median at 23.6 years (18.4–28.6).

In total, 73% (11 of 15) of included studies were judged to have low risk of bias as measured using the Newcastle–Ottawa Scale (Table 1). Reasons for increased risk of bias included uncertain representativeness due to lack of details concerning confirmation of diagnosis of DMD [18], limited comparability due to inadequate description of ventilatory support [12, 13, 18], insufficient follow-up (with respect to the distribution of age in the studied patient sample) [26], and caregiver-reported age at death [13].

Excluding studies with increased risk of bias [12, 13, 18, 26] only had an impact on pooled estimates derived from samples including both ventilated and non-ventilated patients. Specifically, for these mixed cohorts, remaining estimates included the median survival for 43 French patients with DMD born before 1970 (estimated at 25.8 years) and 76 French patients born after 1970 (estimated at 41.0 years), both reported by Kieny et al. [11], as well as the median survival for 67 German patients with DMD born between 1970 and 1980 (estimated at 24.0 years) [19]. The revised pooled median was estimated at 25.8 years (95% CI 24.0–41.0) and revised weighted pooled median at 31.4 years (24.0–41.0).

Including each country only once, the pooled median life expectancy at birth for patients with DMD who did not receive ventilatory support varied between 19.0 and 20.1 years, and weighted pooled median between 19.6 and 20.4 years. Corresponding ranges for ventilated patients were 28.9–30.6 years and 30.6–33.0 years, respectively.

## Discussion

The outcomes of this systematic review and meta-analysis indicate that life expectancy at birth in DMD has improved following advances in standards of care, in particular respiratory management. We estimated the pooled median life expectancy at birth in patients with DMD who did not receive ventilatory support to be 19.0–19.4 years, notably lower than corresponding estimates for patients receiving ventilatory support (29.9–31.8 years). These findings are consistent with reports of causes of death in DMD, which for recent cohorts are more frequently related to cardiac, as opposed to respiratory, involvement [28, 29]. That survival varies between ventilated and non-ventilated groups of patients was also evident from our synthesis of data derived from samples where an undisclosed proportion of patients were ventilated. Indeed, across these cohorts, the difference between the lowest and highest estimate of median life expectancy at birth was 23 years (range: 18.0–41.0).

Looking at differences in life expectancy at birth in DMD over time, only a few studies stratified their sample by year/decade of birth or death (Table 1). However, of those that did, there was a trend of considerable improvement over time. Eagle et al. [9] estimated median life expectancy at birth for UK patients who died in the 1960s and in/after the 1990s at 14.4 and 26.2 years, respectively (the latter with ventilatory support). A relative improvement of similar magnitude was reported by Ishikawa et al. [10] for Japanese patients that died 1964–1984 and ventilated patients who died after 1991 (18.1 vs. 39.6 years, respectively), and to some degree also by Kieny et al. [11] for French patients born before/after 1970 (25.8 vs. 41.0 years, respectively), Passamano et al. [12] for Italian patients born in the 1960s and 1980s (18.0 vs. 28.0 years, respectively), and van den Bergen et al. [13] for Dutch patients born 1961–1974 and 1980–2006 (18.0 vs. 29.0 years, respectively).

It should be noted that synthesized estimates for those ventilated might be biased, as they were derived from patients who had in fact survived up until they received ventilatory support. Consequently, as it cannot be ruled out that mortality in DMD at the population level is elevated also before more pronounced respiratory involvement (even with current standards of care), this sample restriction would be expected to have resulted in an overestimation of life expectancy with ventilatory support in this indication. Additionally, patients who received ventilatory support may also have had access to overall better care, including but not limited to corticosteroid therapy (which have been shown to delay loss of independent ambulation, reduce needs for scoliosis surgery, and improve respiratory function) [14], regular surveillance, and early treatment of any cardiac manifestations of the disease, which may also have contributed to prolonged survival. In other words, it is important to keep in mind that the increased life expectancy reported in more recent research is likely to be a function of a general improvement of the medical management of the disease, including but not limited to the routine use of ventilatory support in advanced stages of the disease. The data analyzed as part of this review do not permit further assessment of the relative contribution of specific care components, but this constitutes a relevant topic for future studies of survival in patients with DMD.

A potential reason for the observed variability in mortality estimates may be related to differences between countries concerning the general medical management of patients with chronic, disabling conditions. For example, in Japan, patients with DMD typically reside in dedicated treatment centers in later stages of the disease, in which they are offered lifelong care and rehabilitation. While institutionalized, these patients would be expected to be closely monitored and treated by healthcare professionals with relevant expertise per current care guidelines. In contrast, in other settings, upon turning 18 years of age, patients instead transition from pediatric to adult services and usually continue to receive the majority of their long-term care at home. We have previously reported on several limitations with this transition process in the UK, where adult patients for example experience less comprehensive care compared to children with DMD, including lower frequency of cardiac and respiratory assessments and access to physiotherapy [30, 31]. Considering the importance of respiratory management noted in this study, harmonizing DMD care across healthcare systems and access points (both within and between countries) appear highly relevant to further improve prognosis for survival in this indication.

Most of the articles included in this meta-analysis were judged to have low risk of bias, and excluding studies with increased risk had little impact on pooled estimates. However, before 1988, there was no certain pathway to diagnosis of DMD (i.e., via DNA analysis) and patients were instead identified via, for example, electromyography, muscle biopsy, and/or creatine kinase levels. Thus, some studies involving older cohorts may have estimated life expectancy for samples also including patients with muscular dystrophies similar to, but different from, DMD, for example Becker muscular dystrophy, or certain types of limb girdle muscular dystrophy. Interestingly, Rall et al. [19] conducted separate analyses of life expectancy for German patients born between 1970 and 1980 diagnosed at the molecular level with a proven out-of-frame dystrophin gene mutation, and for those classified according to other criteria (i.e., identified via a family support group), and noted significant differences in survival between these strata. In particular, as expected given the severity of DMD in relation to other dystrophinopathies, life expectancy at birth was significantly longer for patients without definite DMD diagnosis. Accordingly, in our meta-analysis, retrieved mortality data derived from samples of patients diagnosed prior to 1988, of which few would have had access to ventilatory support, would be expected to overestimate life expectancy in DMD. For this reason, the identified improvement in life expectancy in DMD over time should be regarded as conservative.

The outcomes of this meta-analysis highlight several topics of importance for clinical and health policy. First, because of reductions in mortality from respiratory involvement, treatment of cardiac-related complications, especially cardiomyopathy, is expected to now become one of the most pressing challenges in the medical management of the aging DMD population [4, 28, 29]. Second, given the progressive accumulation of disability and morbidity associated with the disease, as life expectancy improves, additional efforts should be made to help maintain and promote patient quality of life, in particular mental well-being in advanced stages of the disease (where patients approach a state of full paralysis). Third, and last, as patients with DMD live longer, the burden on informal caregivers is also likely to increase, especially in settings where patients primarily are treated at home. It may therefore be relevant to review, and subsequently update, current clinical, social, and financial support schemes directed towards affected families.

A limitation of the present study concerns the fact that we, due to availability of evidence, synthesized median as opposed to mean life expectancy at birth. We had to focus on this measure since not all patients included in research of life expectancy at birth in DMD would be expected to experience the failure event (i.e., die) during follow-up. For this reason, it is not possible to estimate mean survival (calculated as the area under the estimated Kaplan–Meier survival function). Indeed, estimates of mean life expectancy at birth for patients with DMD treated according to current clinical care guidelines (first published in 2010, with revised guidelines released in 2018 [14]) would not be available until the 2060s, since some patients now live into their sixth decade of life [32]. Given that mortality typically is higher in later as opposed to earlier stages of DMD, median survival may overestimate mean survival in this indication. On the other hand, since some patients now live to experience their 50th birthday and beyond, the magnitude of this bias is not easily quantified. We were also, due to absence of data, unable to stratify our pooled estimates by variables known to impact the prognosis for survival in DMD, including corticosteroid therapy. Another limitation with our meta-analysis of medians is related to the fact that we could not formally test heterogeneity (using, for example, I^2^ statistics) as most studies did not report measures of uncertainty. Finally, it is also worth noting that results from one study were limited by a low number of deaths. Specifically, in Kohler et al. [26], only three of 43 patients (< 7%) died during a mean follow-up of 5.4 years, and the majority of patients was censored before the 50% survival time point. Accordingly, the median survival estimate from this study should be interpreted with caution.

In conclusion, median life expectancy at birth in DMD seems to have improved considerably during the last decades. The introduction of ventilatory support into the medical management of DMD appears to have been an important contributing factor to this development. With current standards of care, including cardioprotective management, many patients with DMD can now expect to live into their fourth decade of life.
